# Renal angiomyolipoma with epithelial cyst

**DOI:** 10.4322/acr.2021.308

**Published:** 2021-08-20

**Authors:** Bharti Varshney, Vikarn Vishwajeet, Vijay Madduri, Gautam Ram Chaudhary, Poonam Abhay Elhence

**Affiliations:** 1 All India Institute of Medical Sciences, Department of Pathology, Jodhpur, Rajasthan, India; 2 All India Institute of Medical Sciences, Department of Urology, Jodhpur, Rajasthan, India

**Keywords:** Angiomyolipoma, Neoplasms, Kidney, Nephrectomy, Diagnosis, Differential

## Abstract

Angiomyolipoma with epithelial cysts (AMLEC) is a recently described entity and is an uncommon subtype of kidney angiomyolipomas. AMLEC is a benign entity but usually masquerades a renal cell carcinoma on imaging examination. AMLEC has a distinct histological and immunohistochemical staining pattern, which helps in the pathological diagnosis. We present a rare case of AMLEC in a 26-year-old female, which was provisionally diagnosed as renal cell carcinoma on radiology. We also summarize the differential diagnosis of this rare variant, its characteristic features, and a review of the literature.

## INTRODUCTION

Renal angiomyolipoma (AML) is a common benign triphasic mesenchymal neoplasm comprising variable amounts of thick-walled dysmorphic blood vessels, smooth muscle, and mature adipose tissue.^1^ Due to their triphasic nature, AML was initially considered to be a hamartomatous lesion. Clonality-based studies have now classified AML as neoplastic.^2^ AMLs are usually solid lesions, both radiologically and grossly, so they do not enter into the differential diagnosis of adult cystic renal lesions. An unusual cystic variant of AML has recently been recognized, which poses a diagnostic confusion with adult cystic renal neoplasms. Hence, this rare combination was labeled as angiomyolipoma with epithelial cysts (AMLEC) or cystic angiomyolipoma; the former is more commonly used.^3^^,^ 4 Herein, we report a rare variant of AML with special emphasis on its histological mimickers and how to differentiate them, along with a review of the literature.

## CASE REPORT

A 26-year-old female presented with pain in the right lumbar region and intermittent dysuria for the last 2 years. She had no history of hematuria, awareness of lump, tuberous sclerosis or intake of hormonal supplements. On examination, the abdomen was soft with fullness in the right renal angle and tenderness on deep palpation. However, no lump was palpable.

She underwent a contrast-enhanced computed tomography scan (CECT), which revealed a 12 x 11 cm soft tissue mass involving the upper two-thirds of the right kidney. The mass compressed the right renal vein, sub-hepatic inferior vena cava, and part of the portal vein. Radiologically, it was provisionally diagnosed as renal cell carcinoma (RCC). Her urinalysis showed only occasional red blood cells and urine culture was sterile, and the routine laboratory investigations were within normal limits.

She underwent a right radical nephrectomy. On gross examination, the kidney was enlarged (17 x 11 cm) with a bosselated appearance. The cut surface showed a large solid mass with focal cystic areas (11 x 10 cm) occupying the whole kidney with only small uninvolved renal parenchyma at the lower pole. The solid area was firm, glistening white to focally yellowish. Multiple uniloculated to multiloculated cysts ranging from 1-6 cm in diameter with clear fluid were noted.

**Figure 1:**
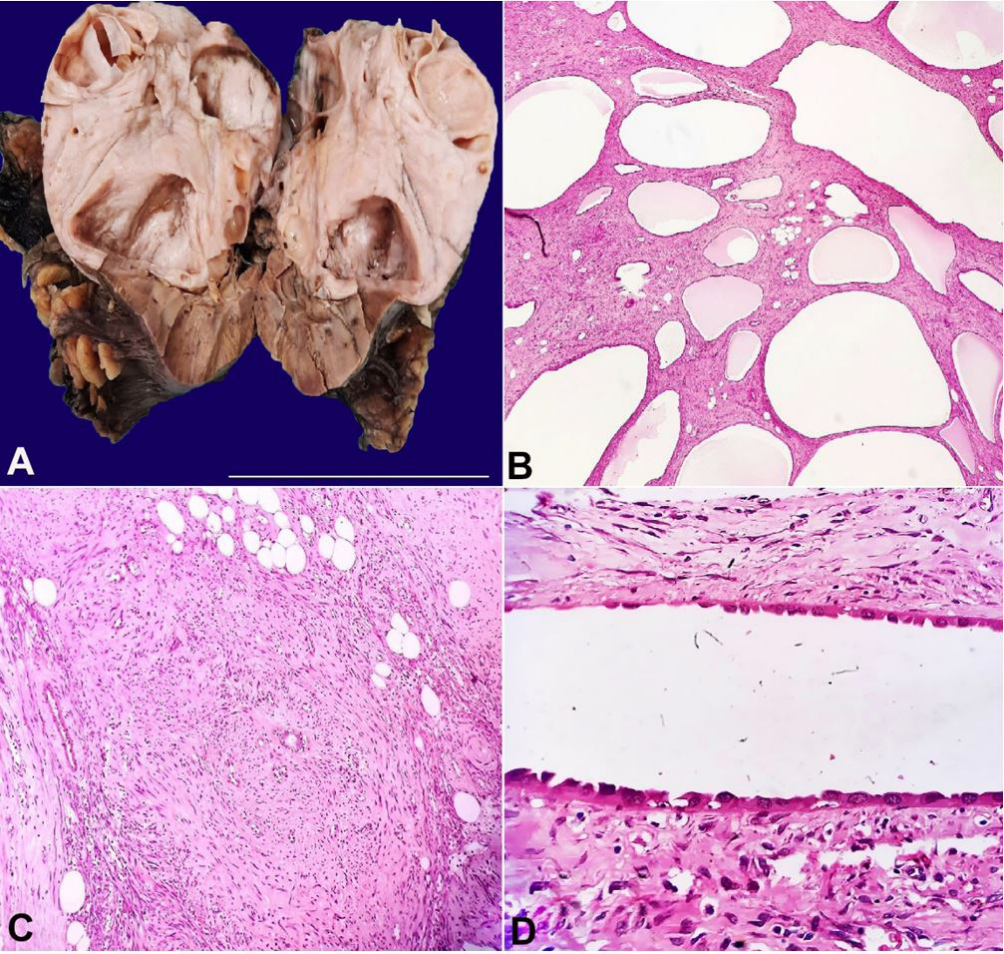
**A** – Gross view of the surgical specimen showing large solid grey white mass with entrapped cystic spaces; **B** – Photomicrograph showing multiple variable sized cystic spaces (H&E, 40X); **C** – Photomicrograph showing hypercellular stroma along with adipocytic aggregates (H&E, 40X); **D** – Photomicrograph showing epithelial cysts lined by cuboidal to hobnailed cells (H&E400X).

The microscopy revealed a biphasic tumor comprised of stromal and epithelial elements. Stroma showed dense collagenization with hypocellular to edematous regions along with few adipocytic aggregates. Focally, the stroma was dense and hypercellular, resembling ovarian stroma. However, no areas of scarring or foci favored ovarian corpora albicantia. Numerous variable-sized thin to thick-walled blood vessels surrounded by proliferating spindle cells were seen with dysmorphic appearance. In stromal cells, no significant cellular atypia, mitosis or necrosis was seen. Variable-sized cystic areas resembling dilated tubules lined by monolayer cuboidal to columnar and hobnailed cells with hyperchromatic nuclei were seen. No blastemal, skeletal muscle differentiation or clusters of clear cells were noted (Figure 1A-D).

**Figure 2:**
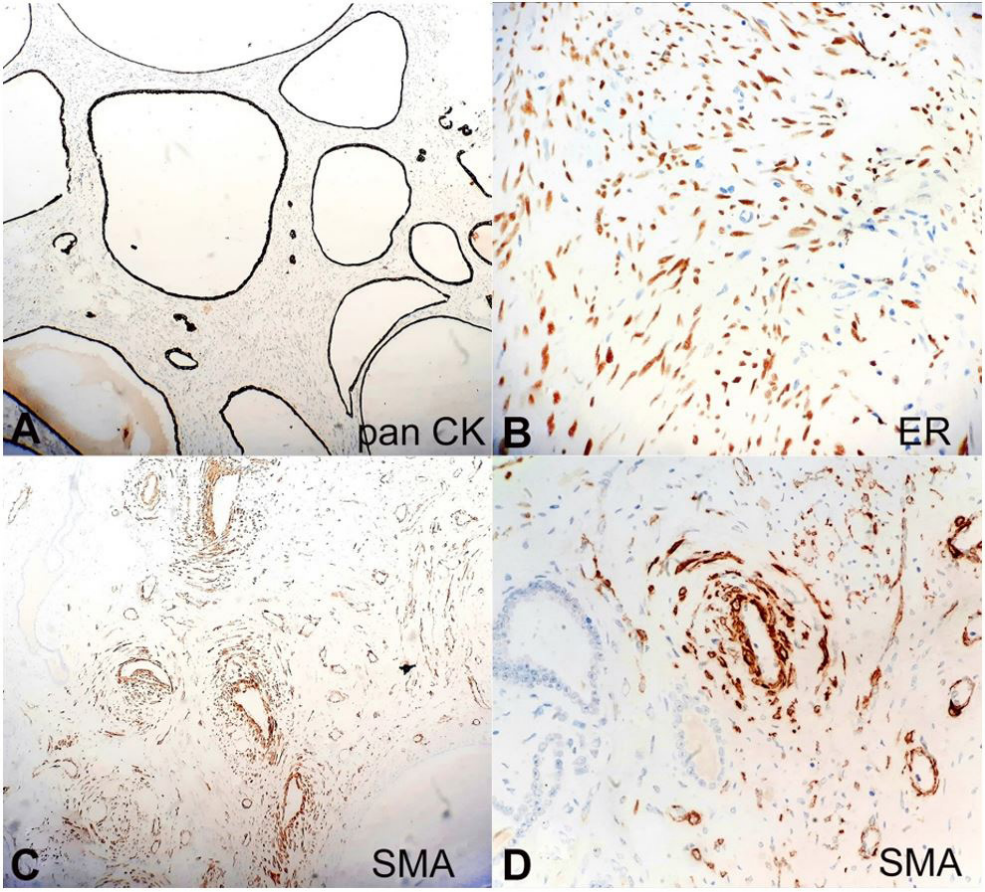
Photomicrographs of the tumor. **A** – Epithelium lining the cystic spaces showing intense pancytokeratin staining (100X); **B** – Compact ovarian like stroma showing nuclear ER staining (400X); **C** – Vessel walls and few stromal cells highlighted by SMA (100X); **D** – SMA highlighting blood vessel (400X).

**Figure 3:**
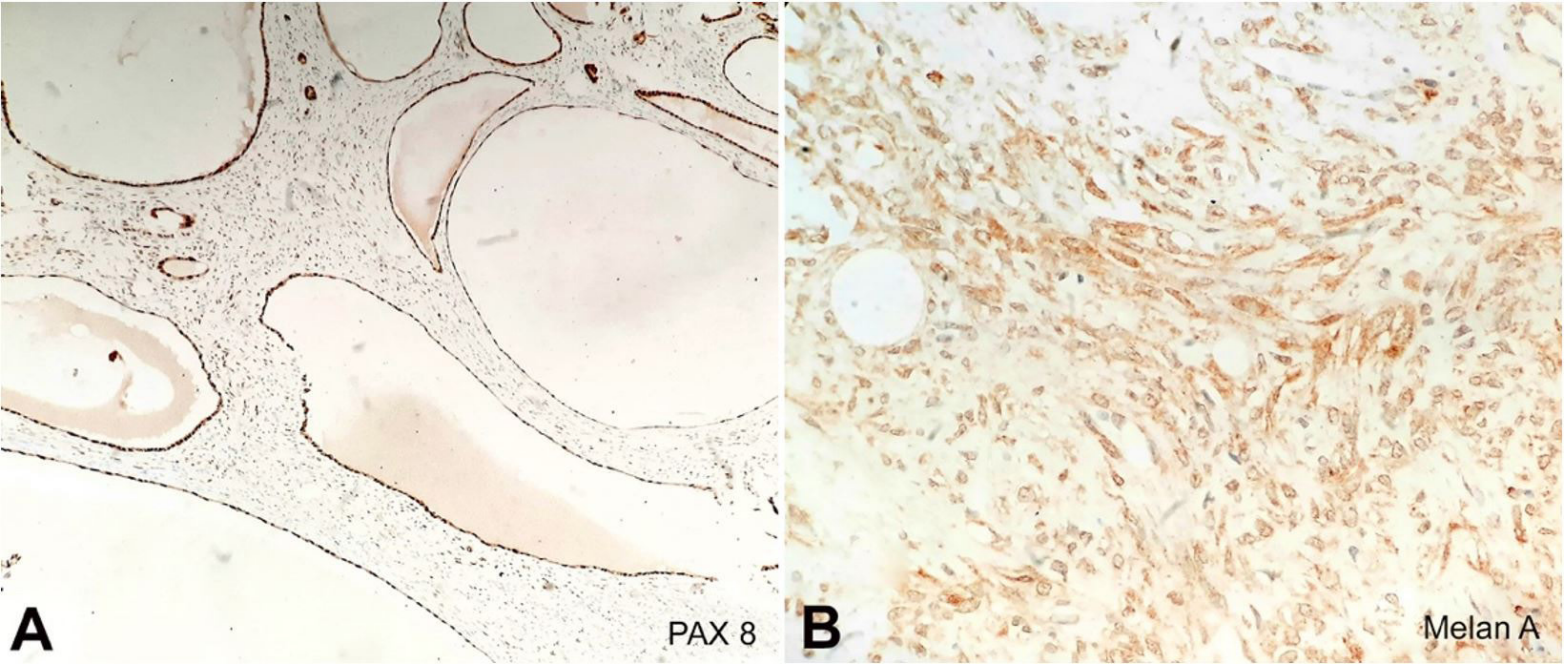
Photomicrographs of the tumor. **A** – Epithelium lining the cystic spaces expressing PAX-8 (100X); **B** – Stromal cells showing diffuse positivity for Melan A (400X).

On Immunohistochemistry (IHC), the epithelial lining of cystic spaces showed positivity for pancytokeratin (CK), PAX-8, dense ovarian-like stroma expressed estrogen-receptor alpha (ER), smooth muscle actin (SMA) highlighted vessel walls and few stromal cells. Diffuse positivity for Melan A, CD10, and HMB45 was seen in stromal cells (Figure 2 and 3).

Thus, a histopathological diagnosis of renal angiomyolipoma with epithelial cysts (AMLEC) was made. At a 6-month follow-up, she is doing well with no complaints.

## DISCUSSION

Renal AMLs are usually solid neoplasms with multiple histological subtypes such as inflammatory, epithelioid, and some with a predominance of either muscle, adipose tissue or vessels. Apart from this, they may have a cystic change, particularly in large-sized tumor due to tumor necrosis or intratumoral hemorrhage. However, renal AMLs with true epithelial cysts have rarely been described in the literature. In 2006, Fine^3^ and his colleagues introduced the term AMLEC. Subsequently, in 2012, the Vancouver classification of renal neoplasia and the International Society of Urological Pathology officially recognized AMLEC as a distinct subtype of AML.^5^ The present case is histologically and immunohistochemically consistent with the diagnosis of AML with multiple epithelial cysts.

AMLEC is a rare variant of AML with cysts and minimal fat tissue. An extensive literature review using combinations of keywords including “renal angiomyolipoma”, “epithelial cyst,” “mesenchymal tumor,” “kidney neoplasm”, “mixed epithelial with stromal tumor”, and “AMLEC” was performed utilizing PubMed (including MEDLINE), Google Scholar and the Cochrane library. A total of 11 publications with less than 30 cases have been previously reported as renal AMLEC, including 11 cases of cystic angiomyolipoma reported by Davis et al.^4^ (Table 1).

**Table 1:** Distribution of previously reported cases of AMLEC

**Author**	**Age(y)/ Gender**	**Radiological findings**	**Procedure**	**Follow up**
Fine^3^	42/M	Suspicious of RCC	Right Radical Nephrectomy	Alive and disease free after 8 years.
	76/M	Bilateral Cystic renal mass	Right Partial Nephrectomy	Alive after 5 years with non-growing cystic mass in left kidney
	55/F	Solid cystic mass in left kidney	Left Radical Nephrectomy	Alive after 1-year post-operative
	37/F	Multiple lesions in left kidney (H/O Tuberous Sclerosis)	Left Radical Nephrectomy	Stable 6 months post-operative
Davis^4^ (n=11)	m45/7F	3 cases showed cystic lesion among 6 cases for which radiology was available	Not available	Available for 5 cases- 4 were alive and disease free after 3years, 9 years, 3 years, 6 months and 1patient died after 3 years due to unrelated disease
	/			
	4M			
Amrah^10^	39/F	Incidentally discovered 2.5 cm complex cystic mass	Left Partial Nephrectomy	Alive and no evidence of recurrence at 12 months
Mikani^11^	55/M	3cm polycystic tumor in left kidney	Left Partial Nephrectomy	Alive and disease free 2 years post-operative
Rosenkrantz^12^	39/F	Solid exophytic mass in left lower pole and thrombus in left renal vein (H/o Lupus Nephritis)	Left Partial Nephrectomy and left vein thrombectomy	Not available
Yeo al 13	67/M	3.5cm exophytic multilocular cystic mass in lower pole of right kidney	Right Partial Nephrectomy	Not available
	75/F	2.8cm size lesion with extrarenal protrusion	Not available	Not available
Karafin^8^ (n=3)	17/M	4.5cm size cystic renal lesion	Not available	Not available
	51/F	1.5 cm size lesion with extrarenal protrusion	Not available	Not available
Wei J^14^	25/F	Multilocular cystic lesion in lower pole of right kidney	Right Partial Nephrectomy	Alive and disease free 18 months after surgery
Wood^15^	33/F	5-6 cm mixed solid cystic lesion in right kidney	Right Nephrectomy	Alive and disease free 9 months after surgery
Gorin^16^	50/M	Complex cystic renal lesions	Partial Nephrectomy	Not available
	53/F			
Wan^17^	41/F	2.5 cm complex cystic mass in left kidney. Cystic RCC vs benign	Right laparoscopic Tumorectomy	No recurrence 8 months post-operative
Our case	26/F	Suspicious of RCC	Right Radical Nephrectomy	Alive and disease free at 6-month

All these reported cases demonstrated similar demographic features as conventional AML, with the age of presentation ranging from 20 - 76 years and female to male ratio of 5:3. Preoperative diagnosis of AMLEC is difficult as it has no characteristic clinical or imaging features. Clinical features are non-specific and include flank pain, hematuria, proteinuria, hypertension, and retroperitoneal hemorrhage. The present case was provisionally diagnosed as RCC on CT scan.

Grossly, AMLEC is a well-demarcated solid tumor with partially cystic structures. Microscopically, it is composed of three elements: epithelial cysts lined by cells with hobnailed appearance, dense sub epithelial Mullerian-like stroma having prominent thick-walled blood vessels, and the presence of a thick muscular wall. These three components exhibit a distinct IHC pattern. Pancytokeratin is expressed strongly in cells lining the epithelial cysts. Subepithelial stroma has strong and diffuse nuclear positivity for ER, PgR, and cytoplasmic positivity for CD10. The muscle component is positive for desmin and SMA. Contrary to typical AML, AMLEC rarely contains adipose tissue, which was also evident in our case.

When dealing with renal grossly cystic lesions, the following possibilities must be considered (i) Adult Polycystic kidney disease (APCKD), (ii) Multilocular cystic Renal cell neoplasm of low malignant potential, (iii) Cystic Nephroma (CN), (iv) Mixed epithelial and stromal tumor (MEST), (v) Cystic partially differentiated nephroblastoma. However, in cases of APCKD, usually, the cysts are bilateral, multiple, lined by cuboidal epithelium, but they lack other elements such as muscle and dysplastic vessels as seen in AMLEC. Moreover, cysts of APCKD cause the destruction of renal parenchyma, eventually leading to renal failure.

Another entity, CN, also contains cystic structures lined by flattened to cuboidal epithelium with hobnailing, separated by thin septae along with sparsely cellular ovarian type stroma. CN lacks thick-walled blood vessels and usually occurs in females.^3^ The multilocular cystic variant of renal cell neoplasm is characterized by multiple cysts separated by delicate septae containing solid nests of clear cells, which are not seen in AMLEC. Lastly, the entity which almost overlaps with AMLEC is the mixed epithelial and stromal tumor (MEST). Clinically, MEST occurs usually in females with a long-term history of estrogen exposure.^6^ Grossly, it contains both solid and cystic areas. Microscopically, the epithelial elements may show a complex architecture of the glands, tubules, and cysts lined by flattened to columnar cells, urothelium, or rarely ciliated cells. In contrast to MEST, the lining epithelium of AMLEC is a single layer of flat, low cuboidal epithelium with hobnailing.^5^ The smooth muscle component in MEST is well developed and usually in the form of well-formed fascicles, in contrast to AMLEC, which has poorly formed smooth muscle fascicles dissected by lymphatic channels.^4^ The vessels of MEST do not show dysmorphic features vis-a-vis AMLEC, which contains vessels of variable thickness and disorganization. Immunohistochemistry helps to distinguish these two entities. AMLEC shows positivity for melanocytic markers such as HMB-45 and Melan A.^7^

The histogenesis of this tumor is debatable. The current view is that the cysts in AMLEC represent entrapped renal tubules that undergo cystic change, or result from neoplastic epithelial differentiation. Fine et al.^3^ favored the former view, which was further supported by Karafin et al.^8^, who illustrated that the cystic epithelial lining in AMLEC expressed PAX2 and PAX8 similar to the renal tubules. On the contrary, Davis et al.^4^ reported that the cystic component was the outcome of true epithelial differentiation, supported by few cases, in which the tumor was entirely outside the kidney and therefore should not have entrapped the renal tubules. This view was also supported by Filho et al.^9^, who described the epithelioid AMLEC, which expressed HMB-45 and Melan A in both stromal part and epithelial cyst lining, thus favoring cysts to be an outcome of neoplastic epithelial differentiation.

Finally, the stroma in AMLEC has Mullerian differentiation with positivity for ER and PgR. This can be explained by the fact that the urinary and genital systems share embryological proximity.^3^ The clinical outcome of AMLEC is not well established. However, no cases of metastasis or recurrence have been documented in the available literature.^3^^,^ 4 ^,^ 10 ^,^ 11

In conclusion, though AMLEC is a rare entity, it should be considered in the differential diagnosis of adult cystic renal lesions. There is considerable morphological overlap between AMLEC and MEST. Therefore, histopathology supported by pertinent immunohistochemistry helps to reach the correct diagnosis.
